# Bile Acids and Their Receptors in Respiratory Diseases: Mechanistic and Therapeutic Perspectives

**DOI:** 10.3390/biom16101497

**Published:** 2026-10-14

**Authors:** Juanjuan Wan, Hui Zong, Dongdong He, Dingtao Li, Jing Yu, Guodong Wang, Liting Liu, Chuansheng Huang, Xinchun Wang, Liping Wang

**Affiliations:** 1 School of Pharmacy, Shihezi University, Shihezi 832002, China; wanjuanjuan@stu.shzu.edu.cn (J.W.);; 2 Department of Pharmacy, First Affiliated Hospital, Shihezi University, Shihezi 832008, China

**Keywords:** bile acids, respiratory diseases, gut microbiota, gut–lung axis, inflammation, immunity

## Abstract

Animal bile has been used for millennia in traditional Chinese medicine as a cough suppressant and bronchodilator. As the primary functional constituents of bile, bile acids (BAs) are believed to mediate these physiological effects. Although the role of BAs in enterohepatic circulation is well-characterized, and recent advances have highlighted their immunomodulatory functions, their pathophysiological significance in the respiratory system has been largely overlooked. This review synthesizes emerging evidence on the multidimensional roles of BAs and their receptor-mediated signaling pathways, with a focus on their contribution to the pathogenesis of major respiratory diseases such as asthma, pulmonary fibrosis, and lung injury.

## 1. Introduction

Bile, a yellow–green viscous fluid synthesized by hepatocytes, exhibits diverse physiological functions and therapeutic potential [1]. Traditional Chinese medicine (TCM) has long harnessed animal bile for medicinal purposes, with sources ranging from mammals (bovine, porcine, bear) to fish, reptiles, and birds, encompassing up to 44 distinct species [2]. Historically, animal bile was used in ancient China to relieve cough and promote choleresis.

Bile acids (BAs) and their salts are the primary functional constituents of bile, mediating most of its physiological effects [3,4]. Beyond their canonical role in emulsifying and absorbing dietary lipids, BAs function as pleiotropic signaling molecules with anti-inflammatory and immunomodulatory properties [5]. For instance, two lithocholic acid (LCA) derivatives, 3-oxoLCA and isoalloLCA, have been demonstrated to regulate T cell differentiation by suppressing Th17 and promoting regulatory T (Treg) cells [6]. Similarly, 3β-hydroxy-deoxycholic acid attenuates immunostimulatory responses in dendritic cells via modulation of farnesoid X receptor (FXR) activity [7]. Collectively, these findings underscore the pivotal role of BAs and their receptors in shaping immune homeostasis, a process intimately linked to respiratory function. This review provides a comprehensive update on the roles and mechanisms of BAs and their receptors in respiratory diseases, aiming to inform future therapeutic strategies and translational applications.

## 2. Synthesis, Metabolism, and Enterohepatic Circulation of BAs

### 2.1. Synthesis and Metabolism of BAs

**Figure 1:**
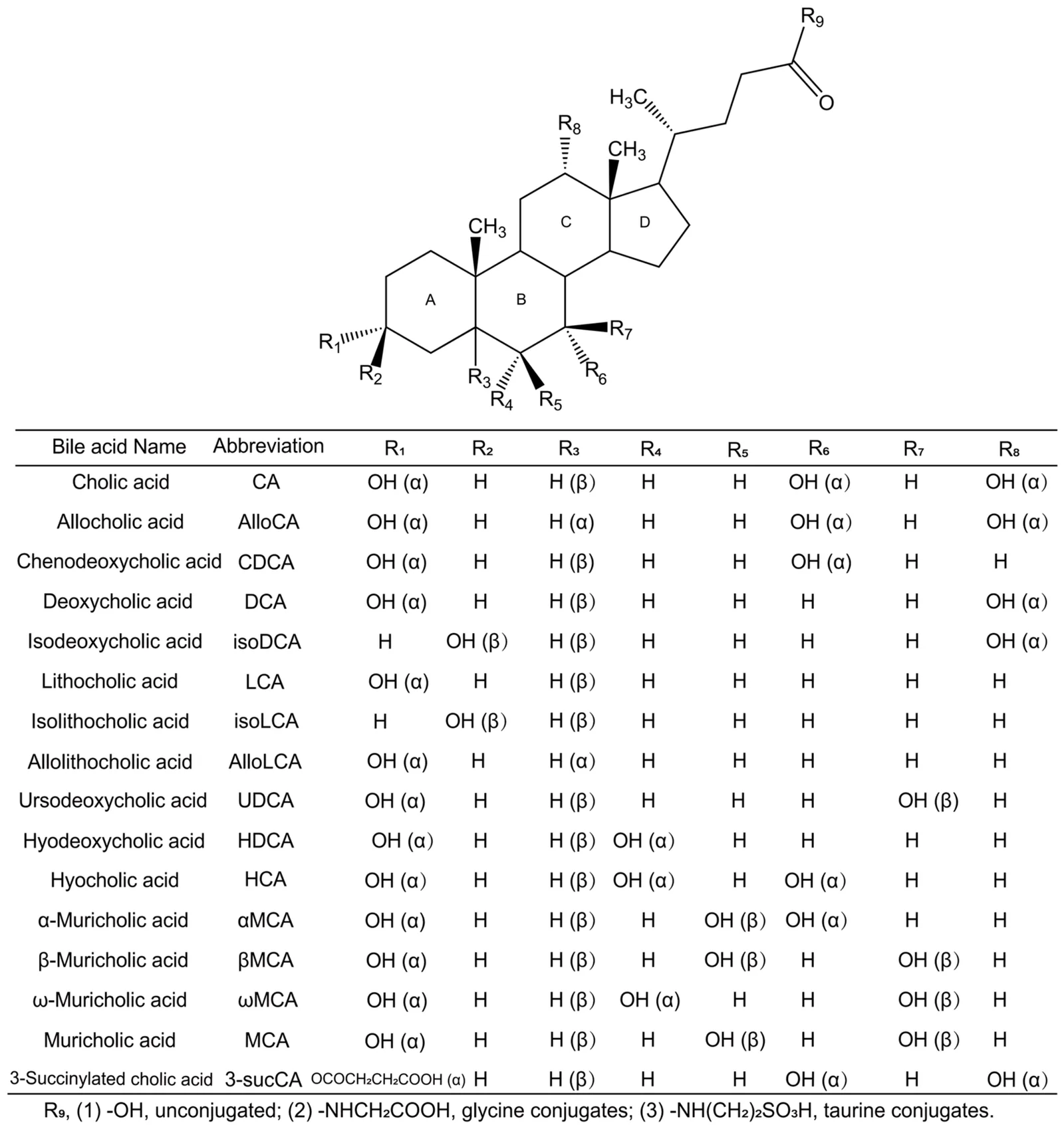
Chemical structures of BAs. Chemical structure and group position of each common BA.

Synthesized from cholesterol in the liver and further modified by gut microbiota, BAs are amphipathic molecules containing both hydrophilic and hydrophobic moieties, conferring detergent-like properties [8]. This structural amphipathicity underpins their classical role in digestion and absorption, as well as their emerging functions as signaling molecules that regulate systemic metabolism and immunity [9]. BA structure varies in side-chain length, carboxylation, hydroxylation, conjugation status, and A/B ring stereochemistry (cis vs. trans), rendering them among the most structurally diverse biomolecules in nature [10,11] (Figure 1).

**Figure 2:**
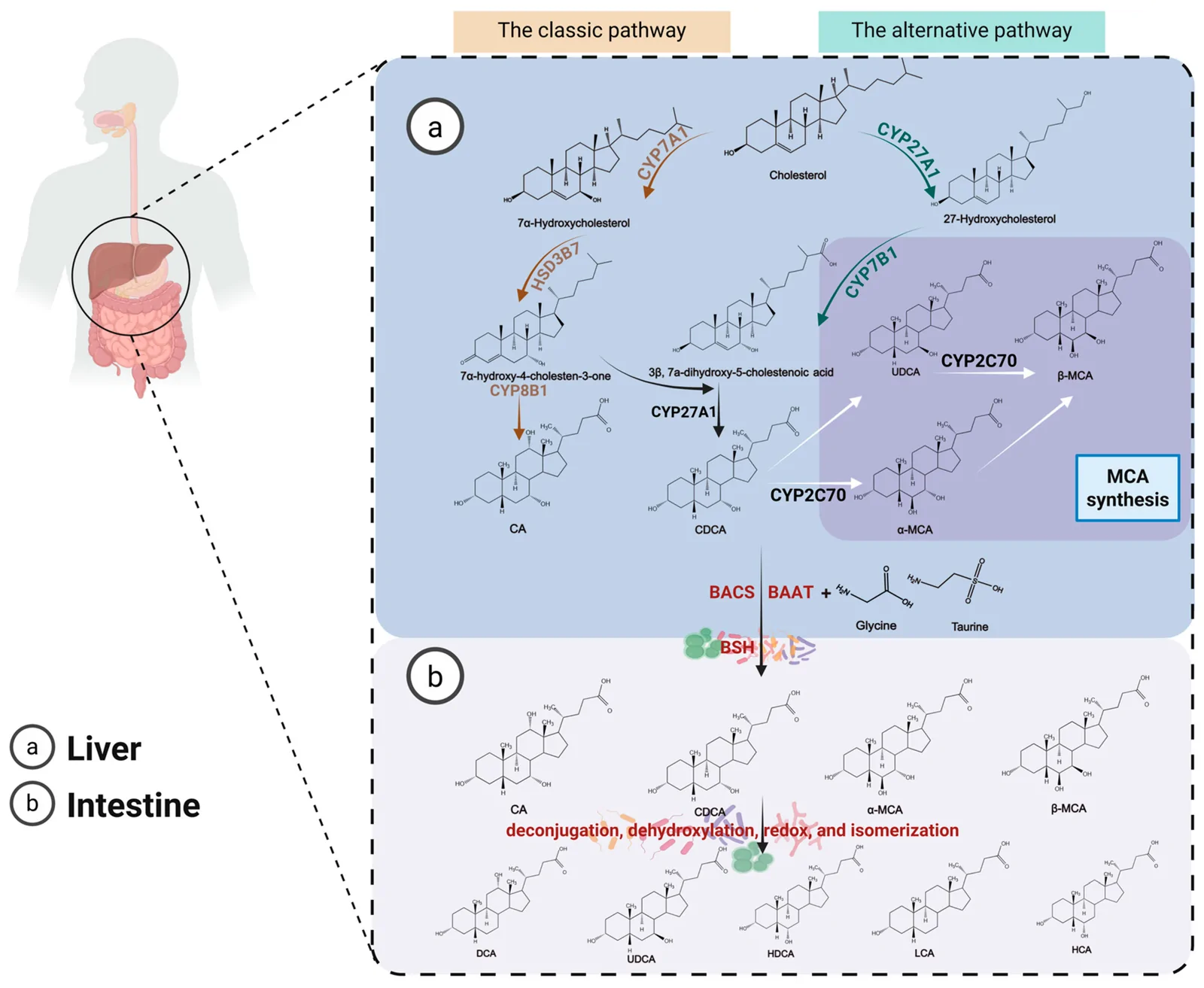
Synthesis and metabolism of BAs in vivo. (**a**) Liver: Cholesterol is converted to primary BAs (e.g., CA and CDCA) via the classical and alternative pathways. (**b**) Intestine: Gut microbiota convert primary BAs to secondary BAs .

BAs are broadly classified into primary and secondary BAs. As illustrated in Figure 2, primary BAs, cholic acid (CA) and chenodeoxycholic acid (CDCA), are synthesized from cholesterol in the liver via the classical pathway and alternative pathways [12]. The classical pathway generates roughly equal amounts of CA and CDCA, accounting for over 90% of total BA production under physiological conditions [13,14]. In rodents, most CDCA is rapidly converted into muricholic acids (MCAs) [5]. Following synthesis, BAs are conjugated with taurine (T) or glycine (G) via bile acid-CoA synthetase (BACS) and bile acid coenzyme A: amino acid N-acetyltransferase (BAAT) in the liver [15], a process that enhances their solubility and reduces toxicity.

In the intestine, Bile salt hydrolase (BSH) deconjugates primary BAs by hydrolyzing glycine and taurine conjugates [16]. BSH enzymes are predominantly expressed in Gram-positive Firmicutes (e.g., *Lactobacillus*, *Enterococcus*) and certain Gram-negative Bacteroidetes, facilitating BA deconjugation and reducing BA toxicity [17]. Subsequent 7α/β-dehydroxylation, catalyzed by enzymes mainly found in *Clostridia* and *Eubacteria*, generates secondary BAs [18]. Gut microbiota also mediate BA oxidation and epimerization via hydroxysteroid dehydrogenases, which reversibly oxidize and reduce the 3α-, 7α-, or 12α-hydroxyl groups [19]. Numerous bacterial species, including *Eggerthella lenta*, *Clostridium scindens*, *Clostridium hiranonis*, *Clostridium hylemonae* [20], *Escherichia coli*, and *Bacteroides fragilis*, participate in BA oxidoreduction [21]. Additionally, *Bacteroides*, *Eubacterium*, and *Lactobacillus* contribute to BA esterification, while *Clostridium*, *Fusobacterium*, *Peptococcus*, and *Pseudomonas* engage in desulfurization [13]. Thus, the gut microbiota profoundly enrich BA diversity and regulate BA synthesis and transport.

### 2.2. Enterohepatic Circulation of BAs

**Figure 3:**
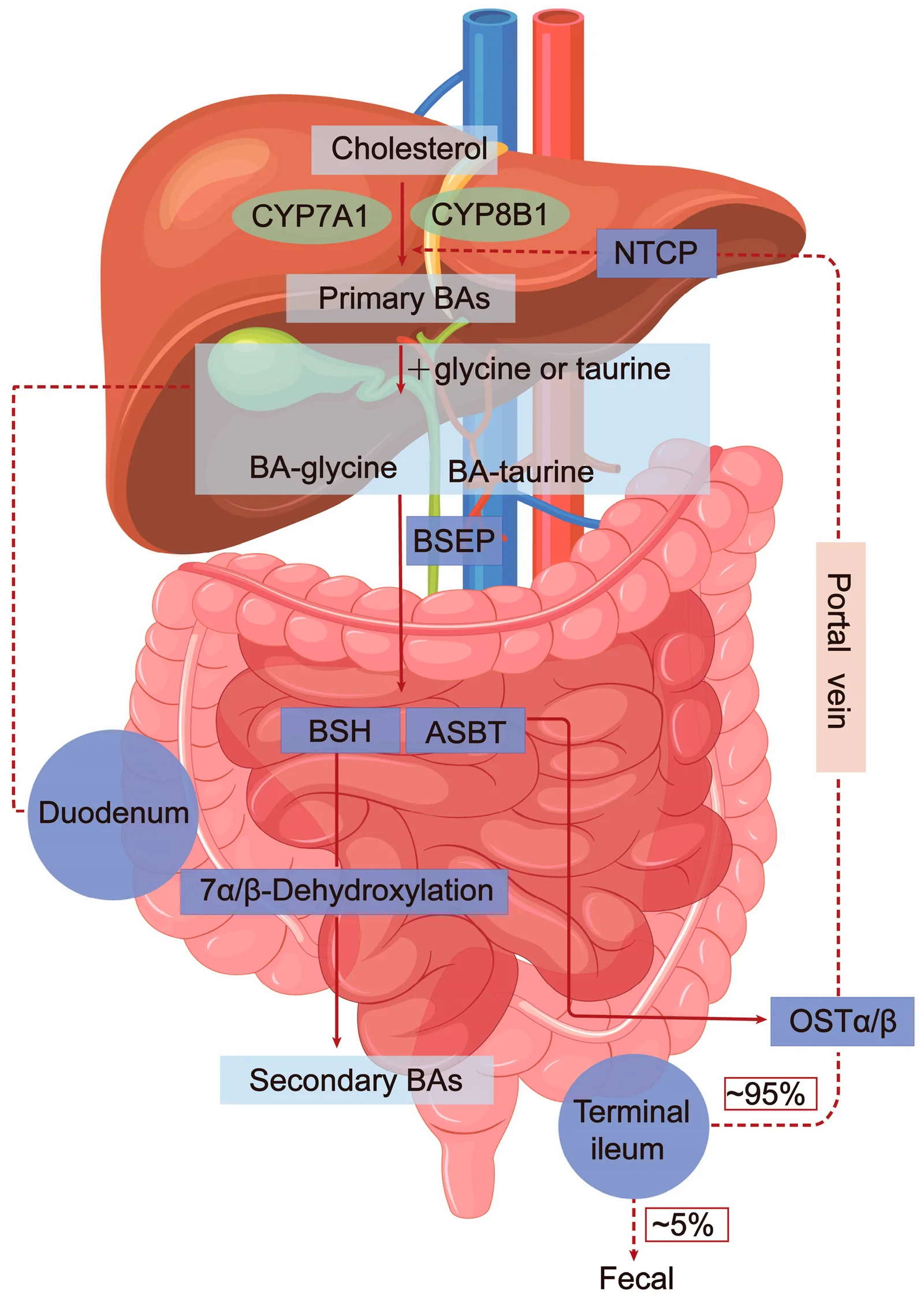
Enterohepatic circulation of BAs. BAs are predominantly secreted by BSEP and subsequently absorbed by the ileal epithelium via ASBT, then transported to the portal vein circulation via OSTα/β. Reabsorbed BAs enter hepatocytes through NTCP and are returned to the liver via enterohepatic circulation. Small amounts of primary BAs also reach the colon, where gut microbiota convert them to secondary BAs. Approximately 95% of the BAs are reabsorbed into the hepatic portal vein, completing the enterohepatic cycle.

BAs synthesized in hepatocytes are actively secreted across the canalicular membrane into bile and stored in the gallbladder. Postprandial release of cholecystokinin triggers gallbladder contraction, releasing bile into the duodenum, where BAs form mixed micelles with dietary lipids to facilitate absorption [22]. The majority of BAs are then absorbed by the ileal epithelium via the apical sodium-dependent bile acid transporter (ASBT) and transported into the portal circulation via the heterodimeric organic solute transporter α/β (OSTα/β) [23]. Upon reaching the liver, BAs are taken up by hepatocytes primarily via the sodium taurocholate cotransporting polypeptide (NTCP) on the sinusoidal membrane, thereby completing the enterohepatic circulation (Figure 3). Approximately 95% of the BA pool is recycled through this pathway, with the remaining 5% (approximately 0.5 g/day) excreted in feces and replaced by de novo hepatic synthesis [22]. The entire BA pool recirculates 4 to 12 times daily, ensuring maintenance of a stable BA concentration and composition critical for normal physiological function [22,24].

## 3. Homeostatic Regulation of BA Metabolism

**Figure 4:**
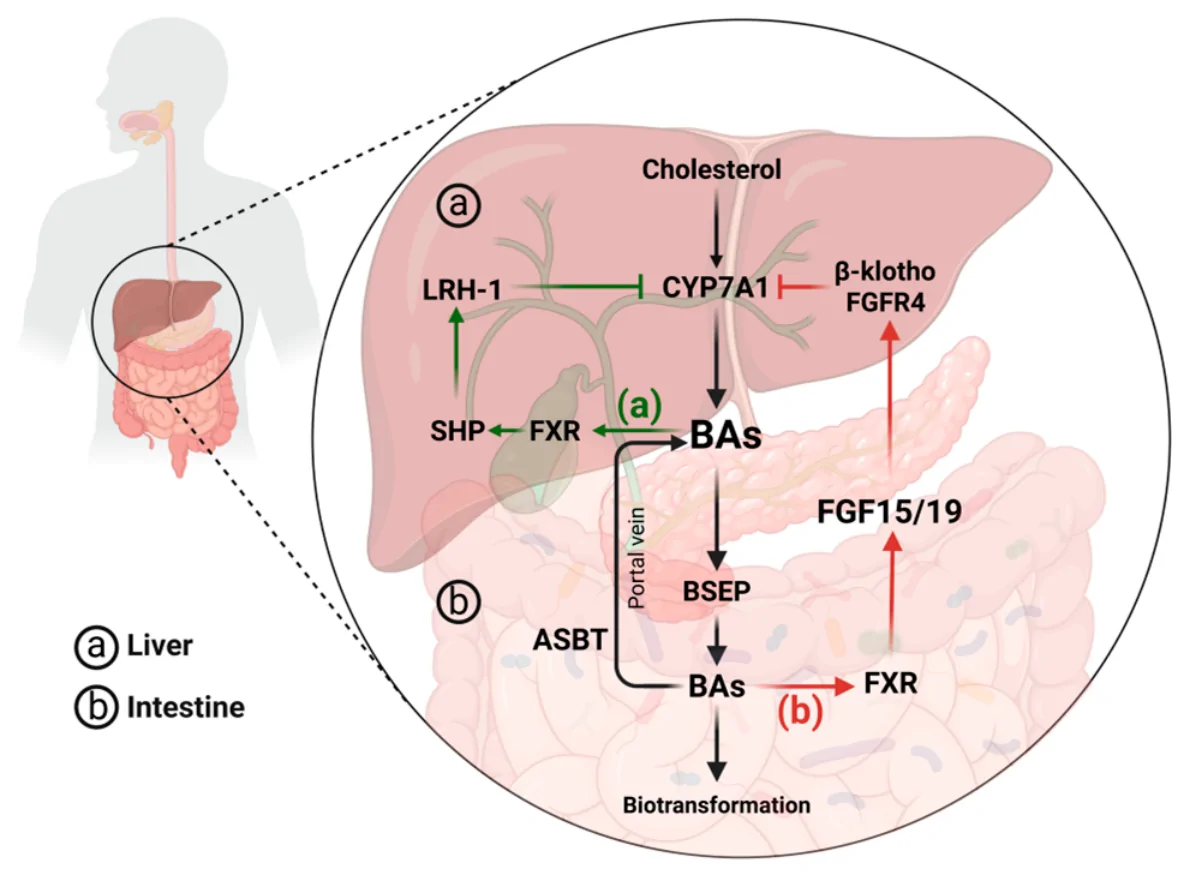
FXR-mediated BA negative feedback regulatory mechanism. (**a**) Liver: FXR-SHP pathway. Increased hepatic BA production enhances FXR-SHP signaling. SHP inhibits CYP7A1 expression by binding to LRH-1, reducing BA synthesis. (**b**) Intestine: FXR-FGF15/19 pathway. BAs are absorbed into the small intestine via ASBT. In enterocytes, they activate FXR, leading to FGF19/15 release. FGF15/19 inhibits CYP7A1 by binding to the FGFR4 β-Klotho complex. Arrowheads indicate promotion, and blunt-ended lines indicate inhibition.

The enzymes and transporters involved in the synthesis, metabolism, and transport of BAs are chiefly regulated by BA receptors [25]. Of these, FXR is one of the most studied receptors. The biosynthesis of BAs is primarily regulated by two negative feedback pathways, both of which involve the activation of FXR [26]. The BAs synthesized in the liver are absorbed into the small intestine by ASBT. In the enterocyte, BAs activate the nuclear FXR, which leads to the subsequent release of fibroblast growth factor (FGF) 19 (or its rodent ortholog FGF15) [27]. FGF15/19 travels to the liver, binding to the fibroblast growth factor receptor 4 (FGFR4) β-Klotho complex and inhibiting CYP7A1 [28]. Meanwhile, increased BA production in the liver promoted hepatic FXR-small heterodimer partner (SHP) signaling [9]. SHP suppresses the expression of the gene CYP7A1 and reduces the production of BAs by binding to liver receptor homolog-1 (LRH-1) [29] (Figure 4). These two pathways are crucial for maintaining BA homeostasis and preventing excessive BA synthesis. However, emerging evidence suggests that local BA receptor signaling in extrahepatic tissues, including the lung, may operate through distinct, tissue-specific regulatory mechanisms that remain poorly defined [30].

## 4. The Gut–Lung Axis as a Target for Pulmonary Disease Treatment

Despite the lung and the intestine having physiological distinctions, the gastrointestinal and respiratory tracts originate from a shared embryonic source and exhibit several anatomical similarities. Recent clinical research has substantiated that gut microbiota significantly influence the etiology and course of respiratory diseases [31,32]. Multiple pulmonary diseases frequently coincide with gut microbiota dysbiosis, potentially linked to disease progression [33]. A mutual regulatory process, or crosstalk, exists between the gut and the lungs, called the gut–lung axis [34].

**Figure 5:**
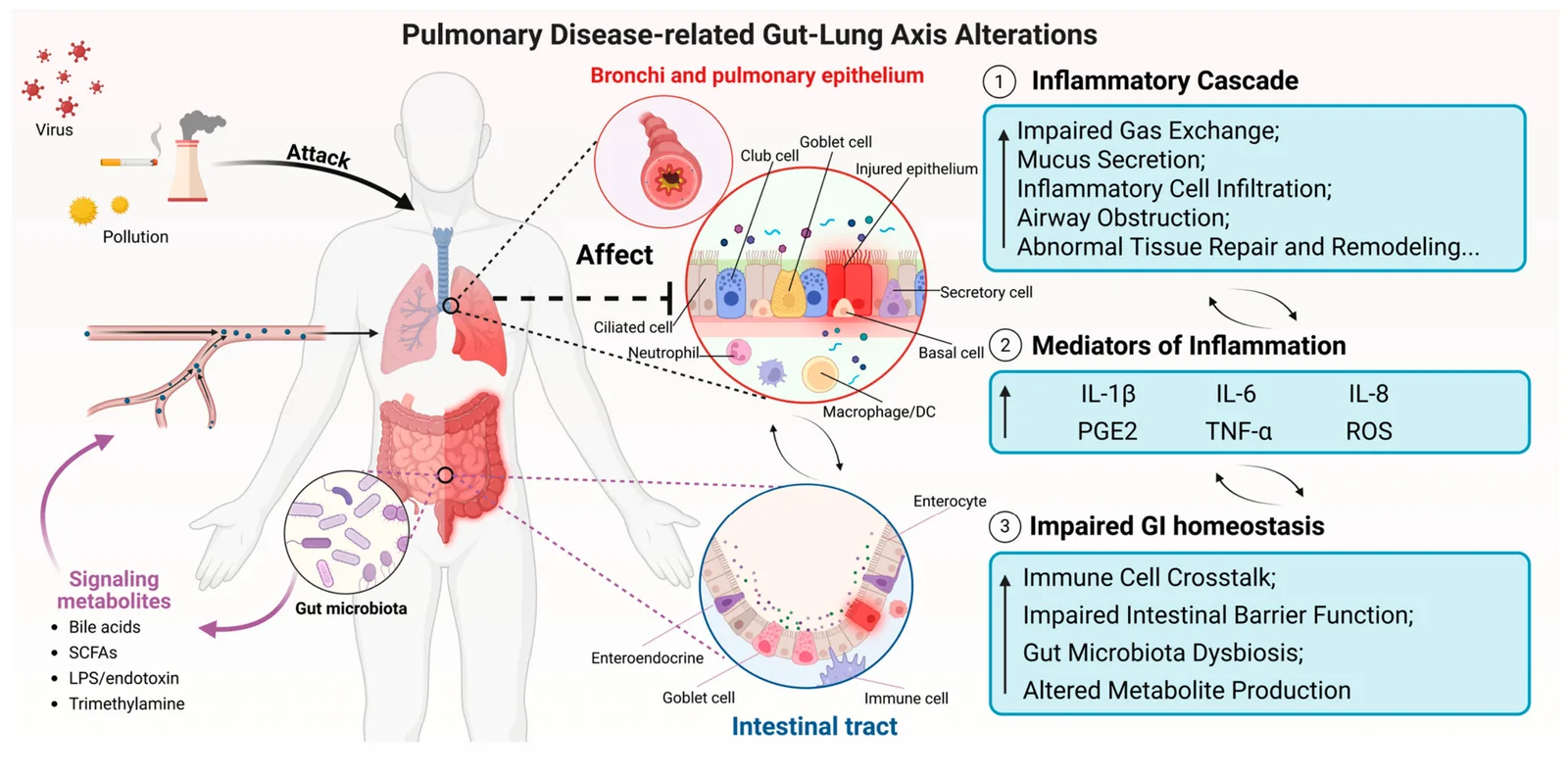
The gut–lung axis: Regulation of pulmonary function via gut microbiota-metabolites. Impaired gut function increases the production and influx of pro-inflammatory mediators into systemic circulation and weakens immune responses, further exacerbating respiratory diseases. Microbiota-metabolites affect lung function through the gut–lung axis and reduce inflammatory responses in the respiratory tract (e.g., bronchial smooth muscle contraction, mucus secretion and inflammatory cell infiltration). Arrowheads indicate promotion, blunt-ended lines indicate inhibition, and dashed lines indicate the magnified regions.

The gut–lung axis suggests that gut microbiota and their metabolites may connect the intestine and pulmonary tissue via the bloodstream and lymphatic system [35], producing bidirectional effects. The gut microbiota may modify metabolic homeostasis, thereby affecting several pathways, including inflammation and immunology, and influencing the progression of pulmonary diseases [36]. Metabolites produced by the gut microbiota, such as BAs, short-chain fatty acids, and lipopolysaccharides (LPS), can influence the pulmonary immune microenvironment via systemic circulation [37,38,39] (Figure 5).

Several gut species have now been linked to BA remodeling in the context of pulmonary pathology. *Clostridiales* and *Eubacterium*, which carry the BA-inducible (*bai*) operon for 7α-dehydroxylation (Section 2.1), generate deoxycholic acid (DCA) and LCA [18]. In a separate line of evidence, DCA and its receptor, Takeda G protein-coupled receptor 5 (TGR5), increase host resistance to influenza virus [40]. *Bacteroides dorei* enhances BSH activity to restore secondary BA production, and the BAs it produces alleviate influenza virus infection in mice via TGR5 signaling [41]. Gut bacteria can also conjugate BAs with amino acids other than glycine and taurine to produce microbial amino acid-conjugated BAs [42], which circulate systemically as host-microbial co-metabolites [43]. In a dietary model, inulin fibre restructured the microbiota-derived BA pool and elevated CA; CA then drove type 2 inflammation at both the intestinal and pulmonary barrier surfaces, dependent on interleukin (IL)-33 and ILC2s. Crucially, knocking out the BSH gene in a single colonizing species (*Bacteroides ovatus*) abolished both the elevation of cholic acid and the pulmonary inflammation [44].

Quantitative profiling studies further demonstrate that the BA pool undergoes measurable remodeling in respiratory diseases. At the systemic level, total BAs were significantly increased in a rat asthma model and negatively correlated with the IFN-γ/IL-4 ratio, suggesting a shift toward T-helper 2 cells (Th2); levels normalized after treatment, paralleling gut microbiota remodeling and improved pulmonary inflammation [45]. In severe COVID-19, primary BA synthesis is selectively depleted, with reduced CDCA and G-CDCA in elderly patients [46]. At the airway level, these changes can be anchored to absolute concentrations: tracheal aspirates from ventilated patients contained 6.2 ± 2.1 versus 1.1 ± 0.4 µmol/L/g of total BAs in those with and without ventilator-associated pneumonia [47], and bronchoalveolar lavage fluid (BALF) from lung transplant recipients reached a median of 1.6 µmol/L (range 0–32) in those who later developed bronchiolitis obliterans syndrome versus 0.3 µmol/L (range 0–16) in those who did not [48]. At the tissue level, data are sparse. Profiling of lung homogenates from obese mice with allergic airway disease revealed selective elevation of βMCA and tβMCA, although the values were presented as relative abundances rather than absolute concentrations [49]. Because circulating BAs are continuously delivered to the lung through the pulmonary circulation, their presence in the pulmonary compartment is expected. What remains to be established is which individual BA species are present, at what concentrations, and to what extent they derive from systemic circulation versus local reflux or aspiration.

Despite these open questions, BAs remain among the most attractive candidates for mediating gut–lung crosstalk, as they are uniquely positioned at the intersection of metabolism and immunity [50]. Their ability to activate specific nuclear and G protein-coupled receptors expressed in pulmonary immune and structural cells provides a direct mechanistic link between intestinal BA metabolism and respiratory health [51].

## 5. Role of BAs and Their Receptors in Respiratory Diseases

### 5.1. Role of BA Receptors in the Pathogenesis of Respiratory Diseases

#### 5.1.1. FXR: A Master Regulator of Pulmonary Inflammation

In the respiratory system, activation of BA receptors can significantly modulate host metabolism and immune function [52]. Among these, FXR is the most extensively characterized target, with accumulating evidence underscoring its pivotal role in modulating respiratory pathologies through immunometabolic crosstalk. TGR5 plays a crucial role in inflammation and immune regulation [53], but more studies are needed to elucidate its mechanism of action and effects in respiratory diseases.

FXR, encoded by the *NR1H4* gene and belonging to the nuclear receptor superfamily, is a BA ligand-activated transcription factor [54] expressed mainly in the liver and gastrointestinal tract but also in non-classical BA-targeted tissues such as the lungs and blood vessels [51].

FXR regulates inflammatory responses: FXR activation in monocytes and macrophages suppresses the toll-like receptor 9 (TLR9)-dependent inflammatory responses, regulates dendritic cell differentiation and inhibits the production of pro-inflammatory factors by natural killer T cells [55,56]. In the LPS-induced mouse model of acute lung injury (ALI), FXR activation can inhibit pro-inflammatory gene expression [57]. In the ovalbumin (OVA)-induced acute rat asthma model, FXR may block the infiltration of inflammatory cells into healthy lung tissue by suppressing the secretion of IL-4, IL-5, and IL-13 from activated Th2 and tumor necrosis factor-α (TNF-α) from activated alveolar macrophages [58], thereby exerting anti-inflammatory effects.

FXR regulates cell fate: Various infections trigger a storm of pro-inflammatory cytokines, including IL-6, leading to diffuse alveolar damage in patients [59]. IL-6 promotes ferroptosis and impairs regeneration of alveolar epithelial type II cells during polyriboinosinic–polyribocytidylic acid-induced murine lung injury by regulating the FXR-PKM2-YAP axis. FXR agonist improves polyriboinosinic–polyribocytidylic acid-associated murine lung inflammation, ferroptosis, and regeneration in mice [60]. In the LPS-induced mouse model of ALI, FXR activation can promote the proliferation of lung microvascular endothelial cells and improve lung permeability and regeneration [57].

FXR regulates viral infection: In the context of coronavirus disease 2019 (COVID-19), the downstream signaling pathways of FXR can regulate the expression of angiotensin-converting enzyme 2 (ACE2), as well as modulate the activation of the immune system and the release of inflammatory factors, which may contribute to the prevention and treatment of COVID-19 [61].

**Figure 6:**
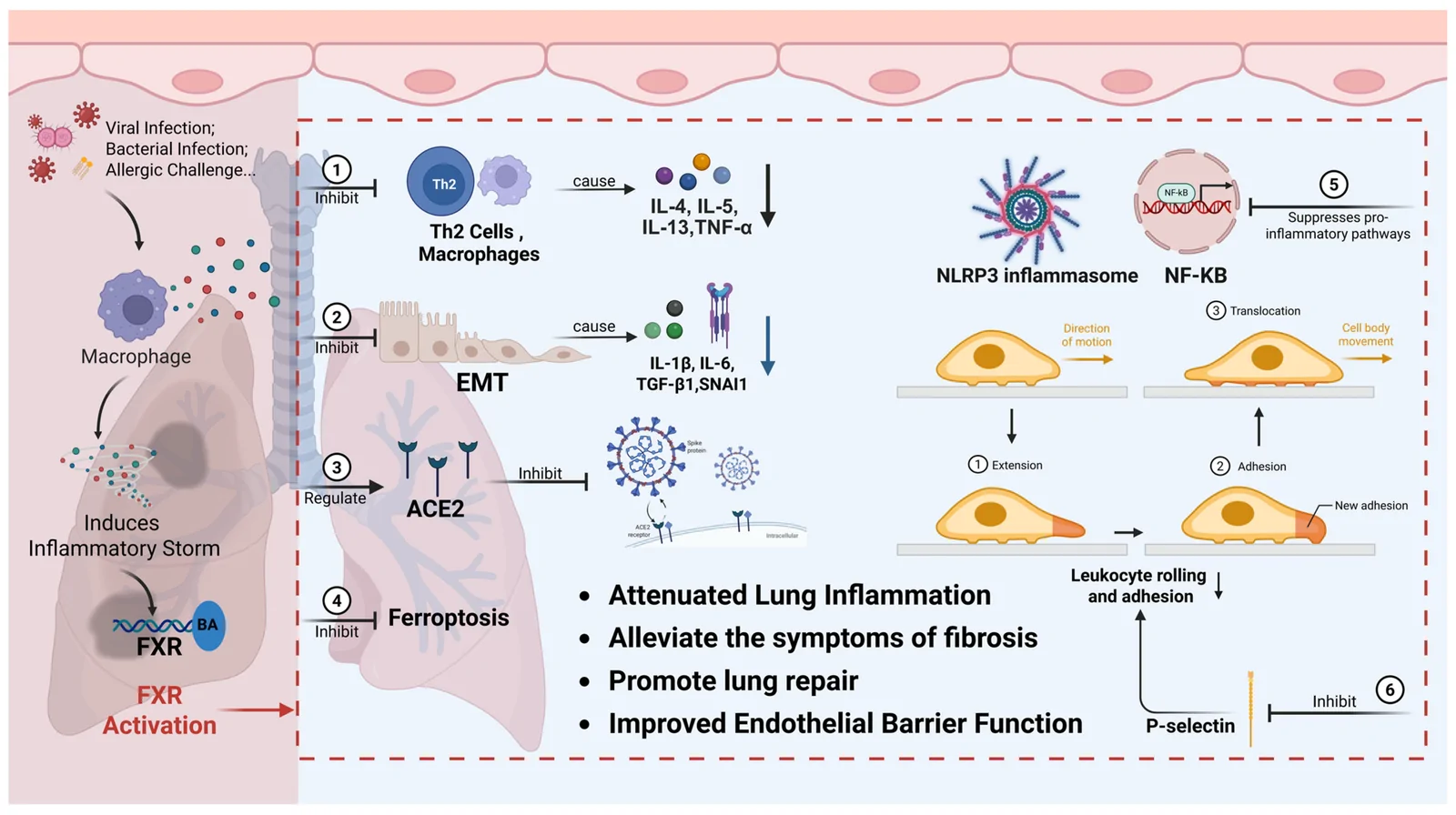
Mechanism of FXR action in lung diseases and its signaling regulatory network. After pathogens invade and cause lung injury, BA activates FXR, which regulates the epithelial–mesenchymal transition (EMT) process and curbs lung fibrosis. Additionally, FXR suppresses pro-inflammatory pathways like NF-κB and NLRP3, activates FOXM1 to boost lung repair factor expression, and adjusts cytokine release. Through these actions, FXR plays a key role in repairing the lungs and fighting inflammation. Arrowheads indicate promotion, blunt-ended lines indicate inhibition. Created in BioRender. Wan, J. (2026) https://BioRender.com/wb217cg.

Thus, through its diverse roles in inflammation, tissue regeneration, and viral infection, FXR can be a biomarker and therapeutic target for these respiratory diseases (Figure 6).

#### 5.1.2. TGR5: Gatekeeper of Immune Tolerance

TGR5, a member of the G protein-coupled receptor family, is expressed in multiple tissues, including the lung [62]. Activation of TGR5 can specifically inhibit the TLR4-NF-κB signaling axis and suppress the expression levels of LPS-induced pro-inflammatory factors, such as TNF-α and IL-6 [63]. TGR5 can also increase the ratio of IL-10 to IL-12 expression by activating protein kinase A signaling, effectively inhibiting the polarization of macrophages towards the pro-inflammatory M1 phenotype [53]. In addition, TGR5 promotes nitric oxide production by phosphorylating endothelial nitric oxide synthase, leading to the downregulation of adhesion molecule expression and monocyte adhesion, thereby hindering leukocyte transport and contributing to the anti-inflammatory effects of TGR5 [64]. It also showed similar effects to FXR in COVID-19 [12]. Overall, activating TGR5 exerts anti-inflammatory and immunomodulatory effects through regulating macrophage M1/M2 polarization and promoting vascular protection, among other mechanisms, highlighting its potential in the treatment of respiratory diseases.

#### 5.1.3. Beyond FXR and TGR5: An Expanding Spectrum of Receptors

FXR and TGR5 are the most extensively studied BA receptors. Other BA receptors can also directly or indirectly influence the physiological functions of the respiratory system [9].

**RORγt**: The microbial secondary BA isolithocholic acid (isoLCA) is a reversible inhibitor of RORγt [65]. Targeting RORγt may therefore offer a therapeutic approach for non-type 2 asthma: in a house dust mite (HDM) model, a RORγt inhibitor blocked both IL-17 and IL-22 as well as airway hyperresponsiveness and neutrophilia, an effect not reproduced by anti-IL-17 alone [66].

**S1PR2**: BAs signal through S1PR2 [67], which is expressed in various lung cell types and regulates inflammatory cytokine release, barrier integrity, and immune cell migration. In an ovalbumin-induced asthma model, pharmacological inhibition of S1PR2 reduced IL-4, IL-5, and IL-13 levels in BALF and attenuated lung inflammation [68,69]. More recently, isoLCA was shown to ameliorate lung inflammation through S1PR2-dependent metabolic rewiring of alveolar macrophages, identifying a microbiota-dependent BA that acts as a selective airway immunomodulator [70].

**Bitter taste receptors (TAS2Rs)**: BAs are physiological ligands for human and mouse TAS2Rs [71]. TAS2Rs are expressed on airway smooth muscle cells, and their activation induces localized calcium signaling that relaxes airway smooth muscle and reverses obstruction in a mouse model of asthma [72].

PPARγ, PXR, and CAR can also bind BAs or modulate BA metabolism, but direct evidence for their involvement in pulmonary BA signaling remains limited. In summary, BA receptors beyond FXR and TGR5 can shape the pulmonary immune microenvironment, although studies in this area remain limited. Whether specific microbial BAs reach their targets and activate these receptors in the lung remains to be determined.

### 5.2. Pathophysiological Roles of BAs in Respiratory Diseases

Building upon the receptor-mediated mechanisms discussed above, this section systematically examines the roles of BAs in specific respiratory diseases. While BA receptor activation generally exerts protective effects, the net impact of BAs in each disease context depends on multiple factors including BA composition, concentration, duration of exposure, and the underlying pathological milieu. The following subsections synthesize current evidence across major respiratory conditions, highlighting both therapeutic potential and, where relevant, pathogenic contributions.

#### 5.2.1. Asthma

Asthma is a common chronic inflammatory airway disease affecting an estimated 260 million individuals worldwide, a number projected to reach 275 million by 2050, driven largely by population growth and modifiable risk factors such as high body mass index, smoking, and air pollution [73]. The disease is characterized by variable respiratory symptoms and airflow limitation, often associated with airway inflammation and remodeling [74]. Emerging evidence suggests that BAs, including those produced via microbial modification, exert protective effects on allergic airway inflammation through multiple mechanisms.

Conjugated BAs, such as tauroursodeoxycholic acid (T-UDCA), significantly attenuate inflammatory responses, mucus metaplasia, and airway hyperresponsiveness in asthma models. These effects are partly mediated by alleviation of the unfolded protein response (UPR) [75]. Specifically, taurohyodeoxycholic acid (T-HDCA) improves allergic asthma by regulating the immune balance of CD4^+^ T cell subsets [76]. Mechanistically, conjugated BAs preferentially interact with activated transcription factor 6α (ATF6α), a key UPR transducer, thereby decreasing allergen-induced UPR activation and resolving allergic airway disease without significantly engaging BA receptors [77]. Clinical relevance is supported by quantitative mRNA analysis of airway epithelial brushings, which demonstrated significant upregulation of multiple ATF6α-related transcripts in asthma patients compared to healthy controls [77]. These findings suggest a novel therapeutic avenue for allergic asthma using select conjugated BAs.

Unconjugated BAs also show therapeutic promise. CDCA ameliorates ovalbumin-induced histological changes by attenuating inflammatory cell infiltration, mucus hypersecretion, and goblet cell hyperplasia [78]. UDCA reduces eosinophilic airway inflammation in sensitized mice via ligation of dendritic cell nuclear FXR [79]. Moreover, UDCA ameliorates airway remodeling, suppresses apoptosis of airway epithelial cells, and dampens Th2 immune responses, collectively contributing to its protective effects in allergic airway inflammation [80].

#### 5.2.2. Pulmonary Fibrosis (PF)

PF is a chronic and progressive pulmonary disease characterized by progressive deposition of extracellular matrix proteins, ultimately leading to respiratory failure and death [81]. Several BAs have demonstrated anti-fibrotic properties in preclinical models.

T-CDCA significantly reduces TNF-α and tissue inhibitor of metalloproteinases (TIMP)-2 expression in pulmonary tissues of fibrotic mice, accompanied by decreased alveolar inflammatory exudate [82]. T-UDCA accelerates recovery from crystalline silica-induced lung injury, an effect linked to reduction of IFN-γ-producing CD103^−^ T_EM_-like and IL-17A- producing CD103^+^ T_RM_-like Treg cells in the lung [83].

EMT is a process by which epithelial cells lose polarity and intercellular adhesion and transition to mesenchymal cells, implicated in bleomycin (BLM)-induced PF [84,85]. In BLM-challenged mouse lungs, both the ERS marker GRP78 and the UPR marker CCAAT/enhancer-binding protein homologous protein (CHOP) were upregulated. T-UDCA pretreatment suppresses the upregulation of GRP78 and CHOP, suggesting that inhibition of ERS contributes to its anti-fibrotic effects [86]. Additionally, T-UDCA attenuates BLM-induced TGF-β1 upregulation and Smad2/3 activation, inhibits α-SMA upregulation, and prevents E-cadherin downregulation, collectively indicating suppression of EMT [87].

Obeticholic acid (OCA), a semi-synthetic FXR agonist and CDCA analogue, protects against BLM-induced lung damage by suppressing EMT, inflammation, and collagen deposition [88]. Notably, OCA administration after PF establishment still significantly improves pulmonary function [89]. OCA also alleviates histopathological changes, oxidative stress, and inflammation induced by nitrogen mustard [90], highlighting its therapeutic potential across diverse fibrotic insults.

#### 5.2.3. Acute Lung Injury (ALI)

ALI and its more severe form, ARDS, are critical clinical syndromes with high morbidity and mortality [91]. ALI is characterized by neutrophil infiltration, alveolar-capillary barrier disruption, diffuse alveolar damage, non-cardiogenic pulmonary edema, and robust local and systemic inflammation, culminating in progressive respiratory failure and hypoxemia [92,93].

**Sepsis-induced ALI.** Sepsis is the most common risk factor for ALI [94]. UDCA alleviates sepsis-induced lung injury by attenuating inflammation, oxidative stress, and pulmonary barrier dysfunction. Mechanistically, these effects are linked to inactivation of the STING pathway and suppression of PANoptosis-like cell death [95].

**Fat embolism syndrome (FES)-induced ALI.** Pre-treatment with UDCA attenuates pathological and biochemical changes in FES-induced ALI [96]. UDCA significantly inhibits inflammatory responses and suppresses TNF-α and IL-1β expression in vitro by inhibiting p38 MAPK/NF-κB signaling and activating FXR [97]. These findings provide new evidence for the therapeutic potential of UDCA in ALI.

**LPS-induced ALI.** CDCA significantly ameliorates lung tissue pathology in LPS-induced ALI mice, modulates cytokine profiles, and alters expression of relevant targets [98]. Both CDCA and UDCA mitigate endotoxin-induced lung injury in rats through modulation of AQP1 and AQP5 expression, reduction of oxidative stress, regulation of apoptotic pathways (BAX, caspase-3, BCL-2), and attenuation of NF-κB-driven pro-inflammatory activity [99]. OCA prevents LPS-induced ALI by enhancing lung FXR activity and blocking phosphorylation of ERK1/2, JNK, p38, and Akt [100].

**Bacterial pneumonia-induced ALI.** DCA exhibits rapid bactericidal activity against *Streptococcus pneumoniae* in vitro and reduces pneumococcal colonization [101]. In LPS-induced ALI, bear bile powder components (T-UDCA and T-CDCA) alleviate lung injury by inhibiting NF-κB activation and reducing CD14 expression [102]. Specifically, T-CDCA reduces pulmonary edema and neutrophil infiltration in *Staphylococcus aureus*-infected lung, downregulates HMGB1 expression, and attenuates inflammatory mediator production through modulation of TLR2 signaling in macrophages [103]. These findings position T-CDCA as a promising candidate for preventing and treating bacterial ALI.

#### 5.2.4. Viral Pneumonia

Viral pneumonia, commonly community-acquired, is caused by influenza virus, parainfluenza virus, respiratory syncytial virus, human metapneumovirus, and adenovirus [104]. BAs have emerged as important modulators of antiviral host defense.

**Influenza.** Secondary BAs act on distal lung tissue by inhibiting NLRP3 inflammasome activation via the TGR5-cAMP-PKA axis, thereby reducing lung inflammation during later infection stages [41]. DCA, through TGR5 signaling, increases host resistance to influenza virus by suppressing virus replication and neutrophil-dependent tissue damage [40].

**COVID-19.** The COVID-19 pandemic, caused by the severe acute respiratory SARS-CoV-2, represents the most extensive global health crisis in a century. Recent evidence links BAs to COVID-19 pathogenesis and treatment [61]. UDCA reduces ACE2 levels in nasal epithelium by inhibiting FXR, thereby decreasing susceptibility to SARS-CoV-2 infection [105,106,107]. Beyond preventing spike-ACE2 binding, UDCA inhibits aberrant airway epithelial cell migration and enhances the restoration of the epithelial basal layer [108]. Derivatives of UDCA also enhance ACE2 exopeptidase activity, suggesting activation of the ACE2/Ang(1-7)/MasR axis to counteract COVID-19-related inflammation [109]. Additionally, CDCA significantly inhibits the activity of Nsp15, a coronavirus uridine-specific endoribonuclease essential for viral replication [110]. Thus, BAs protect against COVID-19 through multiple complementary mechanisms.

#### 5.2.5. Lung Cancer

Lung cancer is the leading cause of cancer-related deaths worldwide, comprising non-small cell lung cancer (NSCLC) and small cell lung cancer (SCLC). Lung adenocarcinoma (LUAD), a major NSCLC subtype, accounts for approximately 40% of cases. Emerging evidence indicates that BAs are also implicated in lung cancer, where they modulate tumor biology and treatment responses through mechanisms that vary with the BA species and the receptors involved.

**Modulation of chemotherapy.** Autophagy may promote resistance to doxorubicin (DOX) in LUAD [111]. UDCA attenuates tumor cell proliferation and modulates MAPK pathway phosphorylation (ERK, JNK, p38), altering Beclin1, LC3B-II/LC3B-I, and P62 expression ratios to diminish DOX-induced autophagy [112]. This suggests UDCA as an adjunct to enhance chemotherapy efficacy.

**Modulation of immunotherapy.** BA metabolism shapes the efficacy of immune checkpoint inhibitors in NSCLC patients [113]. T-LCA enhances anti-tumor immunity by boosting T cell activation and cytotoxic function while modulating immune checkpoints [113]. UDCA dose-dependently suppresses herpesvirus entry mediator expression and slows tumor growth in mouse lung cancer models [114].

**Direct anti-tumor effects.** CDCA inhibits LUAD cell proliferation, migration, and invasion while promoting apoptosis. Mechanistically, CDCA suppresses integrin α5β1 signaling by inhibiting expression of α5 and β1 subunits and phosphorylated FAK, with concomitant increase in p53 levels, a downstream target of this pathway [115]. These findings highlight the therapeutic potential of BAs in LUAD.

**Context-dependent cellular effects.** DCA promotes NSCLC cell migration in a TGR5-dependent manner [116]. TGR5 signaling is itself ligand-biased: in NSCLC cells, the Gs-biased agonist INT-777 inactivates YAP and inhibits proliferation, whereas the β-arrestin 1-biased agonist R399 activates YAP and promotes growth [117]. These findings illustrate the ligand-biased and context-dependent nature of TGR5 signaling in NSCLC.

In summary, in lung cancer, BAs cannot be regarded as a single class of drugs. Whether BAs produce protective or harmful effects may depend on the specific type of BA, the receptors they act upon, and the cellular environment.

#### 5.2.6. Protective Effects of BAs on Other Respiratory Diseases

**Cystic fibrosis (CF).** CF is an autosomal recessive disorder caused by mutations in the CF transmembrane conductance regulator (*CFTR*) gene, leading to defective Cl^−^ channels in mucus- and sweat-producing cells [118]. The lungs are most severely affected, with alterations in airway surface liquid (ASL) increasing mucus viscosity and impairing epithelial function, ultimately compromising mucociliary clearance and bacterial elimination [119]. UDCA stimulates Cl^−^ channels in normal airway epithelium [120], and inhibits transepithelial Na^+^ transport in both normal and CF bronchial epithelial cells without altering transporter expression. The resulting increase in ASL height suggests therapeutic potential for UDCA in CF [121].

**Pulmonary arterial hypertension (PAH).** PAH is a fatal disease characterized by progressive pulmonary vascular remodeling [122]. OCA exerts significant therapeutic effects in monocrotaline-induced PAH, improving exercise capacity and reducing right ventricular hypertrophy and pulmonary vascular remodeling [123,124].

**Pulmonary edema.** In LPS-induced pulmonary edema, UDCA decreases BALF TNF-α/IL-1β levels and lung wet/dry ratios. It stimulates alveolar fluid clearance by upregulating ENaC and Na/K-ATPase [125].

**Rheumatoid arthritis-associated interstitial lung disease (RA-ILD).** RA-ILD represents a severe extra-articular manifestation of rheumatoid arthritis, progressing from chronic inflammation to irreversible pulmonary fibrosis [126]. Through multiple receptor-mediated pathways, BAs modulate innate and adaptive immune responses, regulate fibrosis progression, and exert multifaceted therapeutic effects in RA-ILD [127].

Table 1 systematically summarizes BA-mediated pulmonary pathobiology mechanisms and therapeutic implications.

#### 5.2.7. BAs and the Lungs: Therapeutic Potential Versus Risk of Injury

While BAs hold significant therapeutic potential for respiratory diseases, emerging evidence also implicates them in lung injury under certain conditions. A fundamental mechanism underlying this dual behavior is their direct, receptor-independent interaction with biological membranes. Unconjugated BAs are amphipathic weak acids that partition directly into phospholipid bilayers, where they function as protonophores and ionophores and thereby uncouple oxidative phosphorylation [128]. Mild uncoupling by BAs reduces mitochondrial H_2_O_2_ generation by 30–40% without inhibiting the electron transport chain, an antioxidant action [129]. Conversely, excessive membrane perturbation can induce Ca^2+^-dependent mPTP opening, mitochondrial destruction, loss of membrane potential, and ultimately apoptosis and necrosis [130,131,132]. Notably, individual BAs differ markedly: G-CDCA and T-CDCA permeabilize ANT-containing liposomes, whereas T-UDCA lacks such activity and protects mitochondria [130]. This dichotomy between membranolytic and membrane-protective BAs is a key determinant of their opposing pulmonary effects.

In addition to these direct membrane effects, other mechanisms contribute to BA-associated lung injury. BA aspiration, often secondary to gastroesophageal reflux disease, may also contribute to lung injury [133,134], through interactions with the secretory phospholipase A_2_ pathway, disruption of surfactant function and structure, modulation of inflammatory and local immune responses, and direct cellular toxicity [135]. Through these mechanisms, aspirated BAs disrupt immunity, promote fibrosis, impair cell viability, and trigger inflammatory cascades [136].

In summary, different BAs exhibit distinct mechanisms of action in different tissue microenvironments. This helps explain why BAs have both therapeutic potential and the risk of causing damage. Therefore, the dual role of BAs in lung health and disease necessitates rigorous investigation when developing BA-based therapeutics.

## 6. Conclusions and Perspectives

BAs, traditionally recognized for their role in promoting intestinal lipid digestion and absorption, have emerged as critical regulators of respiratory health and disease. Through binding to specific receptors (FXR, TGR5) and interacting with the gut microbiota, BAs modulate diverse physiological and pathological processes in the respiratory system, including antiviral and antibacterial defense, inflammation, and immune homeostasis.

Despite significant progress, several key questions remain. First, the complex crosstalk among BA receptor-triggered signaling pathways—such as MAPK, NF-κB, and TLR cascades—requires further elucidation and may reveal novel therapeutic targets. Second, understanding the cell type-specific effects of BAs on different immune and epithelial cell populations within the lung will be essential for developing targeted therapies. Third, the gut–lung axis represents an increasingly important framework for studying BAs; integrating microbiota analyses with BA profiling may uncover new mechanisms linking intestinal metabolism to pulmonary outcomes.

From a translational perspective, BA-based therapeutics face substantial challenges in moving from bench to bedside. Current evidence derives primarily from preclinical animal models, with a notable scarcity of reliable clinical data. Key questions regarding optimal dosing, formulation, patient selection, and long-term safety remain unanswered. The dual nature of BAs—protective in some contexts yet injurious in others—underscores the need for rigorous, context-dependent investigation. As new technologies (metabolomics, single-cell sequencing, organoid models) continue to advance, the association between BAs and respiratory diseases will become increasingly clear. With continued research effort, the clinical application of BA-based therapies for respiratory diseases may soon be realized.

## Figures and Tables

**Table 1:** Systematic summary of BA-mediated pulmonary pathobiology mechanisms and therapeutic implications.

Disease	BA Species	Receptor Involved	Key Mechanisms	Therapeutic Effect	Ref.
Asthma	T-UDCA	Not specified	Inhibits UPR and ATF6α	Inflammation ↓, mucus ↓, AHR ↓	[75]
Asthma	T-HDCA	Not specified	Regulates CD4^+^ T cell balance (Th2 ↓, Treg ↑)	Allergic asthma improves	[76]
Asthma	Conjugated BAs	Not required	Binds ATF6α → inhibits UPR	Allergic airway disease resolves	[77]
Asthma	CDCA	Not specified	Inflammatory infiltration ↓, mucus ↓, goblet cells ↓	OVA-induced changes ameliorate	[78]
Asthma	UDCA	FXR	Activates FXR → eosinophilic inflammation ↓	Airway inflammation reduces	[79]
Asthma	UDCA	Not specified	Remodeling ↓, epithelial apoptosis ↓, Th2 ↓	Protective effects in allergic airway inflammation	[80]
PF	T-CDCA	Not specified	TNF-α ↓, TIMP-2 ↓	Alveolar inflammatory exudate ↓	[82]
PF	T-UDCA	Not specified	Pathogenic Treg subsets ↓	Recovery from silica-induced lung injury accelerates	[83]
PF	T-UDCA	Not specified	GRP78 and CHOP suppressed	Anti-fibrotic effects	[86]
PF	T-UDCA	Not specified	TGF-β1/Smad2/3 ↓, α-SMA ↓, E-cadherin ↑ (EMT ↓)	BLM-induced PF attenuates	[87]
PF	OCA	FXR	EMT suppressed, inflammation ↓, collagen ↓	Protects against BLM-induced lung damage	[88]
PF	OCA	FXR	Not specified	Pulmonary function ↑	[89]
PF	OCA	FXR	Pathology ↓, oxidative stress ↓, inflammation ↓	Nitrogen mustard-induced injury alleviates	[90]
ALI	UDCA	Not specified	STING inactivated; PANoptosis suppressed	Inflammation ↓, oxidative stress ↓, pulmonary barrier dysfunction ↓	[95]
ALI	UDCA	Not specified	Inflammatory responses inhibited	Pathological and biochemical changes attenuate	[96]
ALI	UDCA	FXR	p38 MAPK/NF-κB inhibited → TNF-α ↓, IL-1β ↓	Anti-inflammatory effects	[97]
ALI	CDCA	Not specified	Cytokines and targets modulated	Lung tissue pathology ameliorates	[98]
ALI	CDCA/UDCA	Not specified	AQP1/AQP5 modulated; oxidative stress ↓, NF-κB ↓; apoptosis regulated (BAX/caspase-3 ↓, BCL-2 ↑)	Endotoxin-induced lung injury mitigates	[99]
ALI	OCA	FXR	FXR activity ↑; MAPK/Akt signaling blocked	LPS-induced ALI prevented	[100]
ALI	DCA	Not specified	Rapid bactericidal activity	Pneumococcal colonization ↓	[101]
ALI	TUDCA/TCDCA	Not specified	NF-κB inhibited; CD14 ↓	Lung injury alleviates	[102]
ALI	T-CDCA	TLR2	Edema ↓, neutrophil infiltration ↓, HMGB1 ↓; TLR2 modulated	Bacterial ALI prevented and treated	[103]
Viral Pneumonia	Secondary BAs	TGR5	NLRP3 inhibited via TGR5-cAMP-PKA	Lung inflammation during later infection stages ↓	[41]
Viral Pneumonia	DCA	TGR5	TGR5 activated → viral replication ↓, neutrophil damage ↓	Host resistance to influenza virus ↑	[40]
Viral Pneumonia	UDCA	FXR	FXR inhibited → ACE2 ↓	Susceptibility to SARS-CoV-2 infection ↓	[105,106,107]
Viral Pneumonia	UDCA	Not specified	Aberrant migration ↓; epithelial repair ↑	Protective effects beyond spike-ACE2 binding	[108]
Viral Pneumonia	UDCA derivatives	Not specified	ACE2 activity ↑ → ACE2/Ang(1-7)/MasR axis activated	COVID-19-related inflammation counteracts	[109]
Viral Pneumonia	CDCA	Not specified	Nsp15 inhibited	Viral replication ↓	[110]
Lung Cancer	DCA	Not specified	Proliferation ↓; MAPK modulated; autophagy markers altered → autophagy ↓	Chemotherapy efficacy enhances (adjunct to doxorubicin)	[112]
Lung Cancer	CDCA	Not specified	Integrin α5β1/FAK suppressed; p53 ↑	Proliferation ↓, migration ↓, invasion ↓; apoptosis ↑	[115]
Lung Cancer	UDCA	FXR	Inhibits FXR → HVEM expression↓	Tumor growth↓	[114]
CF	UDCA	Not specified	Cl^−^ channels stimulated	Therapeutic potential for CF	[120]
CF	UDCA	Not specified	Na^+^ transport inhibited; ASL height ↑	Therapeutic potential for CF	[121]
PAH	OCA	Not specified	Not specified	Exercise capacity ↑; right ventricular hypertrophy ↓, pulmonary vascular remodeling ↓	[123,124]
Pulmonary Edema	UDCA	Not specified	TNF-α ↓, IL-1β ↓, lung wet/dry ↓; ENaC ↑, Na/K-ATPase ↑ → fluid clearance ↑	Pulmonary edema ameliorates	[125]
RA-ILD	BAs (general)	Multiple receptors	Immunity modulated; fibrosis regulated	Multifaceted therapeutic effects in RA-ILD	[127]

## Data Availability

No new data were created or analyzed in this study.

## Abbreviations

BAs, bile acids; T, taurine; G, glycine; CA, Cholic acid; CDCA, Chenodeoxycholic acid; CYP7A1, cytochrome P450 family 7 subfamily A member 1; CYP27A1, cytochrome P450 family 27 subfamily A member 1; UDCA, Ursodeoxycholic acid; MCA, Muricholic acid; BACS, bile acid-CoA synthetase; BAAT, bile acid CoA: amino acid N-acyltransferase; BSEP, bile salt export pump; MRP2, multidrug resistance-associated protein 2; ASBT, apical sodium-dependent bile acid transporter; OSTα/β, organic solute transporter α/β; NTCP, sodium taurocholate cotransporting polypeptide; BSH, bile salt hydrolase; FXR, farnesoid X receptor; TGR5, takeda G protein-coupled receptor 5; FGF15/19, fibroblast growth factor 15/19; FGFR4, fibroblast growth factor receptor 4; SHP, small heterodimer partner signaling; LRH-1, liver receptor homolog-1; DCA, Deoxycholic acid; LCA, Lithocholic acid; HDCA, Hyodeoxycholic acid; SCFA, short-chain fatty acids; ALI, acute lung injury; OVA, ovalbumin; OCA, obeticholic acid; PF, pulmonary fibrosis; BLM, bleomycin; NSCLC, non-small cell lung cancer; COVID-19, coronavirus disease 2019; ACE2, angiotensin-converting enzyme 2; HDM, house dust mite; ARDS, acute respiratory distress syndrome; FES, fat embolism syndrome; SARS-CoV-2, severe acute respiratory syndrome coronavirus 2; Nsp15, nonstructural protein 15; DOX, doxorubicin; CF, cystic fibrosis.
